# Registered nurses’ experiences of communication with patients when practising person–centred care over the phone: a qualitative interview study

**DOI:** 10.1186/s12912-020-00448-4

**Published:** 2020-06-19

**Authors:** Eva Boström, Lilas Ali, Andreas Fors, Inger Ekman, Annette Erichsen Andersson

**Affiliations:** 1 Department of Nursing, Umeå University, Umeå, Sweden; 2 Institute of Health and Care Sciences, Sahlgrenska Academy, University of Gothenburg, P.O. Box 457, 405 30 Gothenburg, Sweden; 3 Centre for Person-Centred Care (GPCC), University of Gothenburg, Gothenburg, Sweden; 4 Närhälsan Research and Development Primary Health Care, Region Västra, Götaland, Sweden

**Keywords:** Person-centred care, Professional role, Telephone, Qualitative

## Abstract

**Background:**

To explore registered nurses’ (RNs’) experiences of practising person-centred care (PCC) by telephone with people diagnosed with chronic obstructive pulmonary disease and/or chronic heart failure.

**Methods:**

Qualitative interview study. Four RNs were individually interviewed before, during, and after participating in an intervention practising PCC by telephone. The interviews were analysed using qualitative content analysis.

**Results:**

The results reflect three categories of their experience: realize the complexity of practising PCC by distance, gain insight into what PCC communication meant to RNs and their approach, and develop the professional role by practising PCC theory and ethics.

**Conclusions:**

PCC over the telephone facilitate healthcare and support patients. Through careful listening, the RNs (1) created space for the individual patients to express their thoughts and feelings and (2) emphasized each patient’s capabilities and resources. The RNs also gained an understanding of PCC and what it means to patients and to themselves as practitioners. Potential implications are that it is important for RNs practising PCC by telephone to remould their role, to listen carefully, and to communicate as equals in conversations that respect both parties’ knowledge and expertise. Health professionals need supervision and support to fully understand the person-centred approach and provide communications that support it.

## Background

With increased life expectancy, the non-communicable chronic diseases that often accompany ageing, and limited financial and human resources [1, 2], a worldwide need exists for new ways to deliver healthcare. Computerized health systems (eHealth), which include telehealth (video or telephone conversations) [3] can provide sufficient care to some populations and have been suggested as a promising solution [1]. People with chronic illnesses such as chronic obstructive pulmonary disease (COPD) and chronic heart failure (CHF) have a high symptom burden and require frequent hospitalization [4]. COPD is currently the fourth leading cause of death worldwide and is predicted to be the third by 2020 [5]. Telehealth in terms of phone support has been reported to be efficient and manageable for patients with CHF [6] and deemed relevant for people living with COPD [7]. Telephone support in general has been described by RNs to be characterized by careful listening, holistic assessments and thereto handle stressful communication [8, 9]. Research have found that telephone support includes ethical dilemmas due to talking through a third party [10] and that RNs have to recompense for the lack of visibility [11]. A review examining person-centred phone support indicated a positive impact on patients’ health-related quality of life [12] and a more recent study showed that telephone support significantly reduced depression symptoms and increased self-efficacy among people with COPD [13]. Registered nurses’ (RNs) provision of home support by telephone for people with CHF has been reported to allow patients a more equal role in care planning and decision making [14] and a more active role in their self-management [15].

Person-centred care (PCC) is based on a relationship of mutual respect and equality [16, 17]. This relationship is fostered in health care by professionals’ use of active listening to patients, considering the patients’ narratives along with results from medical tests and examinations as the basis for jointly formulated health care plans. Patient narratives help professionals identify patients’ internal and external resources, expectations, possibilities, and barriers [16, 17]. PCC for patients with CHF has been evaluated in several studies and shown to be both beneficial from the patients’ perspective [18, 19] and cost-effective [20]. PCC requires a fundamental change in care that emphasizes mutual respect between professionals and patients and recognition of ethical–moral behaviour [16]. Previous research by the authors in PCC showed that professionals remould their position as medical experts [16, 21], and go beyond usual care to co-create individual care with each patient, reflecting both of their perspectives [22, 23]. Telehealth can be a way to improve care for people with chronic illnesses. Such communications, conducted according to PCC values, could make patients more equal and active partners in their own care. Telehealth provides promising new ways to deliver healthcare, but how RNs practice PCC in clinical work, especially at a distance by telephone, is still largely unknown. This study will contribute to an understanding of how RNs respond to and interact within an intervention employing PCC over the telephone.

## Methods

### Aim

The aim of this study was to explore RNs’ experiences of practising PCC over the telephone with people diagnosed with COPD and/or CHF.

### Design

This qualitative descriptive interview study focused on RNs’ experiences of practising PCC by distance was a sub-study within a larger randomized controlled trial (RCT) Care for Ourselves [24].

### Participants and recruitment

A convenient sample of four RNs, all women, employed at a research ward at Sahlgrenska University Hospital/Östra, Gothenburg, Sweden and, all of them who were working in the C4 intervention project, were asked to participate in this study. All received oral and written information and gave written consent to participate. The RNs had 19–30 years of clinical work experience and had participated in workshops concerning person-centred care. The interviewer had earlier knowledge in research and interviewing, and no earlier relations with the participants.

### Setting in the intervention

In total, 221 patients with COPD and/or CHF participated in the RCT (103 intervention, 118 control). Both groups received usual care according to treatment guidelines [4, 5]. In addition, the intervention group received PCC by telephone, provided by four RNs who worked exclusively with the PCC intervention. These RNs received training about COPD and CHF and in PCC theory [16, 17]. Training was also provided in PCC communication (e.g., active listening, asking open-ended questions; reflecting; and summarizing) through lectures, seminars and workshops provided by researchers from various areas (e.g., PCC, nursing, medicine, pedagogy, and communication). The RNs also participated in follow-up booster sessions every other week throughout the study period [16, 25] to discuss completed calls from a theoretical and practical perspective. During the study period (Jan. 2015–Nov. 2016) the RNs conducted 332 telephone calls (mean 3.22 per patient, range 1–10), out of which 309 were called by the RNs and 23 by the patients.

The patients’ mean age was 77.6 years; 54.3% were women; 56.6% lived alone and 43.4% were married or lived with a partner; 49.3% were diagnosed with COPD, 39.8% with CHF, and 10.9% with both. Four patients died before their first scheduled telephone contact. The frequency and duration of the calls were directed by the wishes and needs of the patients and lasted a mean total of 81.6 min (median 70.0 min, range 0–378 min). The RNs shared responsibility for all calls unless a patient expressed the desire to be contacted by a specific RN. During the calls the RNs were asked to maintain an open perspective and invite patients to discuss whatever they wished about their illness. A PCC communication guide including suggestions of topics (e.g., symptoms, life-style, illness, feelings, resources, environment, and social network) was used as a tool for the RNs to encourage people to manage their illness and discover their own solutions for satisfactory self-care.

### Data collection

The four RNs who performed the intervention were individually interviewed three times during the study period: before, during, and at the end of the intervention. One of the RNs, who changed jobs later in the study period, was interviewed only once, before the intervention. All 10 interviews were digitally recorded and conducted by the first author in a quiet room on the research ward.

Semi-structured interviews including questions about their experiences, pre-understandings, perceptions, and views were gathered to get a full impression of their experiences of providing PCC by telephone (see Supplementary file 1, for translated Interview Guide). All interviews started with an open question that encouraged participants to narrate their experiences, such as “Would you please tell me about your experiences of being a part of this intervention of practising PCC by phone?” and “What does person-centred care mean for you? How do you work to achieve a person- centred care? Can you describe opportunities and difficulties in working with person-centred care? Do you have a different approach today after participating in this intervention?” The same interview guide was used at the three different time points in order to capture their thoughts and reflections over the intervention period. The interviews ranged between 41 to 72 min (average 55 min).

### Analysis

The interviews were transcribed verbatim and analysed using qualitative content analysis [26]. First, the interview data was read and listened to several times by the first and last author to get a sense of the whole. In the next step the text was divided into meaning units comprising words, sentences, or phrases responding to the aim of the study. Thereafter, each meaning unit was coded and categorized using Open Code 4.03 [27]. Through further abstractions, three categories were formulated, each including two or three subcategories. As the eligible participants were limited to four RNs, the collected data were carefully reviewed by the authors, and assessed to meet the aim of the study. To reach trustworthiness, throughout the analysis all authors had ongoing discussions and compared the codes, subcategories, and categories, with the original text until consensus was reached. The interpretation of the text was carried out by the first author but critically reflected upon and validated by co-authors. All authors participated in drafting the manuscript.

## Results

**Fig. 1:**
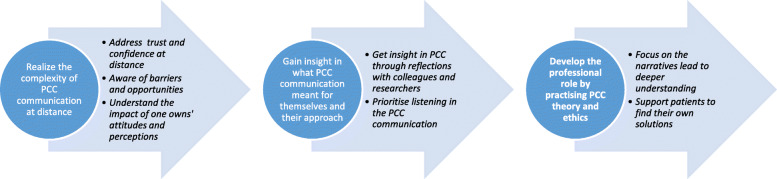
The process of developing a new professional role through reflection on theory and practice

The findings indicate that, over the course of the intervention, the RNs achieved a deeper understanding of how PCC can be applied over telephone, and that their professional role was developed. In the first category the RNs; *Realize the complexity of PCC communication at distance*, in the second they; *Gain insight in what PCC communication meant for themselves and their approach*, and in the third they; *Develop their professional role by practising PCC theory and ethics*. Each category and the various subcategories are presented in Fig. 1.

### Realize the complexity of PCC communication at distance

The RNs realized the complexity of practising PCC by telephone since they had only the patients’ voices and words to rely upon, and no facial expressions or eye contact. The RNs had to *receive trust and confidence at a distance*, they became *aware of barriers and opportunities*, and finally, they understood *the impact of one’s own attitudes and perceptions* in PCC communication.

#### Receive trust and confidence at distance

Each conversation was experienced as a unique rendezvous during which patients discussed their situation and thoughts with trust and confidence. The depth of the conversations was somewhat unexpected for the RNs, showing the patients’ need and desire to share their life experiences with someone who listened. The stories were seldom about the disease per se, and questions about medication and treatment were unexpectedly rare. The communications mainly concerned the patients’ lives and existential issues, such as whether and how they would be able to live with dignity and content or fulfil even humble dreams in the face of severe illness. The RNs were surprised that communication with chronically ill patients worked well without the need for face-to-face consultation, and that they were given trust and confidence. *“Talking to each other by phone goes well. The patient knows I do not know her and did not meet her at the hospital. So maybe that’s why she opened up and confided in me. Then, in some way it may also be a bit easier for me to speak by telephone [not knowing each other]”.*

#### Become aware of barriers and opportunities

The RNs revealed a growing awareness of barriers and opportunities in the PCC communication such as how dependent one was on having an image of the person, including body language and facial expressions. The RNs mentioned that in face-to-face meetings, it was always possible to put a hand on the patients arm for comfort. Lacking such confirmative gestures over the phone, the RNs realized that it was necessary to sharpen other communication skills, such as active listening. They found that they encouraged the patient to talk about what was meaningful for them instead of structuring the communication according to a predefined agenda. The RNs became aware that PCC by distance had its advantages, such as the positive effect on the patients of being at home, where they could feel safe and secure when telling their stories. “*For some people, it seems that talking to a stranger without any physical contact is actually comforting*”. The RNs thought this could be a reason for the rich content in the patients’ stories.

#### Understand the impact of one’s own attitudes and perceptions

In the process of developing awareness of their own attitudes and contributions to the communications, the RNs found that traditional ways of providing information were seldom satisfying to either themselves or the patients. The RNs realized that they were just a brief visitor in the patients’ whole life situation and how surprisingly easily they fell back into well-known patterns such as presenting medical “truths” or traditional information and recommendations without taking the patient’s own resources and knowledge into account. In the beginning, the RNs found it difficult to hold back their urge to speak and to allow patients to talk about what they wanted to focus on. When the RNs reverted to traditional information-giving, they could feel how they lost the patients’ attention. *“I can also feel when they disappear a bit and do not fully pay attention to [what I am saying] and I think*. *.*. *if this supportive conversation is going on in an advisory tone or if I speak constantly ... I can almost feel how they look through the window and listen a bit polite but they goes out there”.*

### Gain insight into what PCC communication meant for themselves and their approach

The RNs gained insights into what PCC by telephone meant for themselves and to their approach towards the patients. The RNs got *insight into PCC through reflections with colleagues and researchers* and they started to *prioritize listening in the PCC communications*.

#### Gaining insight into PCC through reflections with colleagues and researchers

Through dialogue and conversations with the team (colleagues and researchers), the RNs scrutinized some of their telephone conversations that had been tape-recorded and transcribed verbatim. Working according to person-centred values initially raised confusion and discussion among the RNs about whether they should change their professional approach or if this was an approach they were already using? By sharing their difficulties and strategies, they were able to build consensus about how unprejudiced and supportive PCC conversations ought to be performed. The RNs emphasized that PCC was more than having the right approach or meeting the person with respect. It was a deliberative movement from seeing *what* a person is to seeing *who* this person is (i.e., adopting a holistic point of view, considering the patients’ resources and capabilities, and being aware of their possibilities and potential hindrances in order to establish a partnership in care. The values and ethical considerations that influenced the encounter were also apparent in the interviews. “*It’s an attitude, it’s an ethics, and it’s a philosophy: any kind of reason that you base your practice on, and it does not come immediately.”*

#### Prioritize listening during the PCC communication

The RNs gained new insights when they realized that they could contribute their knowledge and advice humbly and according to the patients’ requests rather than to their own agendas. The RNs described that by changing the way that the questions were asked, e.g. by involving “*how”, “in what way”* or “*would you be able to do something to change your situation?”* they were able to take a more equal position and the communications became more vivid and respectful. Avoiding closed questions was found to be especially important, as was making confirmatory listening noises (“mhmm”) so the patient felt heard. Having only a voice to relate to, however, was difficult and demanded experience and training. Listening meant taking the time to reflect upon what the people expressed in their tone of voice and mood, as well as through their words, and it included allowing silent pauses to give the patient space. “*I have been working for 25 years in care and I have never listened the same way I do now.*”

### Develop the professional role through practising PCC theory and ethics

The RNs’ professional role developed through their practising PCC ethics and *focusing on the patients’ narratives, which led to deeper understanding of the patients as persons* and thereby *supporting patients to find their own solutions.* This remoulded their position from being “the expert” to being a partner willing to listen and share expertise in the communication.

#### Focusing on the narratives led to deeper understanding of patients as persons

The RNs focus on listening led to a deeper understanding of their patients as persons and for their individual situations. The in-depth stories narrated by patients focused often on their resources and capabilities, but also on existential questions about how to keep on living. According to the RNs, “*It was fascinating to hear all the stories, including those of fulfilling dreams despite the patients’ severe illnesses”.* Sometimes the RNs were told private secrets and felt there was a fine line between PCC communication and therapeutic conversation, and tried to steer patients away from overly personal issues. The RNs were clear that respect for the patient also included knowing when to use soft control to direct the stories and keep conversations on track by asking open questions about managing their illnesses in daily life, which encourages patients to come up with their own strategies and solutions. The RNs gave feedback and summarized the conversations at the end, but sometimes also during the conversation to confirm that they had understood the patients correctly. The RNs would then excerpt the patients’ story, document its essence in a health plan, and make it accessible to the patient.

#### Supporting patients to find their own solutions

The RNs said that supporting and helping the patients discover their own solutions was the essence of the communication. Supporting patients also meant remoulding the professional role. It was vital in the RNs’ view that the relationship confirm patients as experts on their own lives and themselves as partners in their care who could contribute knowledge and recommendations according to the patients’ requests. It was important to remould their professional role and apply a mindful ethical perspective in the conversation, which included taking care not to provide redundant advice or unrequested information and focusing on, and empowering, the patients’ own abilities and resources. “*When I’m in the conversation now, I think … I feel professional and I have my own skills, but I do not come and force them on the patients. I try to take up their own resources and strengthen them”.*

## Discussion and conclusion

### Discussion

This study explored RNs’ experiences of communicating by telephone to practise PCC with people diagnosed with COPD and/or CHF. The RNs described going through a process from realizing the complexity of telephone versus face-to-face communications, gaining insight into PCC, and finally developing their professional role though shifting their focus to the patient’s own agenda. In contrast to earlier research discussing RNs telephone support and communication [8], discussing how practising PCC by telephone means remoulding the professional role to encourage patients to participate more actively in their own care and building a partnership, will give further perspectives of challenges with telephone support.

#### Remoulding the professional role

The findings suggest that the RNs changed their ways of thinking about their own role as RNs and this affected their ways of interacting with patients at a distance. Drawing knowledge from theories on learning and change, the remoulding of the professional role can be discussed in relation to three interacting facilitating mechanisms: [1] The *learning sessions* provided by the research team, inducing constant reflection and self-observation that resulted in awareness of how previously internalized patterns of practice could hamper PCC support and partnership [2];. The *situated learning experiences* [28] guided the RNs to practise and test different ways of thinking and interacting with patients; and [3] The formation of a small *community of practice* [29] among the participating RNs provided a safe space where they could reflect upon and discuss their experiences. This trajectory towards a remoulded professional identity seemed to be an iterative process of going back and forth between previous assumptions and new ways of thinking, which was not always easy for the RNs and created a substantial feeling of insecurity. Previous research shows that a professional role provides a nurse with a set of assumptions about the content and meaning of the nurse–patient relationship, which creates a necessary psychological security that enables everyday work [30, 31]. Thus, challenges to basic assumptions can prompt what Schein calls “learning anxiety” and if this anxiety is not handled correctly, the process of change will be impeded [30] p.303). The ability to provide psychological safety to reducing learning anxiety has been found to be crucial to professional change [32–34]. Further studies are needed to underpin if and how these facilitating mechanisms, can be used and refined to implement PCC conversations in different settings.

#### Partners in care

The results showed that when RNs gained insight into the ethics of PCC they changed their attitudes and approaches towards patients. They were attentive listeners, asked open questions, and related to the patient’s perspective in contrast to traditional information-giving in which health care professionals set the agenda. Other studies are in accord with our results and have shown that RNs have a leading role and considerable power in their interactions with patients; when RNs use open questions, reflections, and summaries in their communications with patients, they increase the patients’ chances to participate in the dialogue [35]. A positive attitude towards patients’ participation [36] and focusing on patients’ situations and conditions are important factors to enable active patient participation in care and collaboration between the parties. When patients are encouraged to take the initiative, their participation in care increases, they become better prepared to manage their situation [37], and their ability to self-manage their chronic illnesses improves. A supportive healthcare environment is necessary to promote patient participation, and healthcare professionals have a crucial role in making the partnership that is a vital aspect of PCC ( [16, 38] possible in clinical care [39, 40]. A person-centred perspective requires relational ethics “to aim for the good life, with and for others in just institutions” [41] as a basis for health care [16, 17]. Such ethics serve as a guide to facing the moral dilemmas we see constantly in every situation with patients and relatives. Human capabilities can be noted or neglected, strengthened or diminished, especially in situations characterized by asymmetric relationships such as in health care. The RNs in the present study developed their sensibilities to be aware of patients’ needs, but even more of their human capabilities and wishes to remain autonomous and to share their experiences with professionals. The starting point in PCC is the ethics, which recognize that each person is unique. Even when diagnoses and treatments are determining aspects of care, when the patients’ needs and resources are identified and included, care and communication can be tailored to treat them as *who* they are, not *what* their diagnosis is.

### Strengths and limitations

Even though the interviews were conducted among a quite small number of informants, all eligible RNs that performed the intervention were included in the study. Moreover, each RN were interviewed three times during the study period: before, during, and at the end of the intervention. In this way is was possible to capture the participants process of reflection, learning and their experiences throughout the study. The interviews were regarded as rich in content and answered the aim of the study. Qualitative content analysis [26], is an appropriate method when elaborate on informants’ experiences and was applicable to the collected data. Criticism to this analysis method has been raised that there may be a risk if the text is broken into pieces, it may change the overall meaning [42]. To overcome this, the whole text was read several times by two authors so the overall meaning of the text maintained in focus during the analysis. For getting a structural analysis the program Open code 4.03 [27] was used. To achieve trustworthiness, such as dependability and credibility, the analysis has been performed with the help of all authors. According to transferability a humble approach must be considered due to the small sample and that this is a single site study. The interpretation of the text is presented with categories and sub-categories in a figure describing the process of professional development (Fig. 1). The findings are illustrated by representative quotations in the text to help the reader to understand and assess the trustworthiness of the analysis.

### Conclusion

PCC by telephone can facilitate the health care of patients with chronic illnesses. RNs need to include patients as equal partners in their care and thereby start a process towards a remoulded professional identity. Emphasizing each individual’s abilities, capabilities, and resources has been shown to be successful, however, health professionals need supervision and support themselves to fully comprehend the person-centred approach and communicate in such a way that the patient can gain insight into their own capacities to manage their daily lives.

### Practice implication

When practising PCC by telephone, it is important for RNs to remould their role, listen carefully, and communicate as equal partners to allow the parties’ different knowledge and expertise to be fully considered. The relevance of this study can be transferred to other contexts where PCC by telephone can facilitate health professionals’ communication according to PCC values and ethics to support patients with respect and encourage their active participation.

## Supplementary information

**Additional file 1.**

## Data Availability

The data set generated during the study will not be shared due to participants’ anonymity and confidentiality and to respect for the participants’ sensitive contribution. Even though the data are with the corresponding author; the data generated, used and analysed during the current study but are available from the corresponding author on reasonable request.

## Abbreviations

CHF: Chronic heart failure
COPD: Chronic obstructive pulmonary disease
RCT: Randomized controlled trial
RN: Registered Nurse
PCC: Person-centred care
