# Supplementary material for: Registered nurses’ experiences of communication with patients when practising person–centred care over the phone: a qualitative interview study
Source: BMC Nurs. 2020 Jun 19;19:54. doi: 10.1186/s12912-020-00448-4 (PMC7304080; doi:10.1186/s12912-020-00448-4)
Supplement: Supplementary file 1 — Additional file 1. [file 12912_2020_448_MOESM1_ESM.docx]

Supplementary file 1

**Interview Guide**

The semi structured interview guide includes five topic areas;

***1) Working experiences as a nurse?***

*Example of probing questions;* Can you tell us about your experiences as a nurse?

***2) Person-centered care?***

*Example of probing questions;* Can you tell us what person-centered care means to you?

***3) The relationship with the patient?***

*Example of probing questions;* Can you tell us what relationship you have with patients during supportive / PCC calls?

***4) The role of yourself and the patient?***

*Example of probing questions*; Can you tell us how you experience your own role in the meeting with the patient during supportive / PCC calls?

***5) Telephone Support?***

*Example of probing questions;* Can you tell us what experiences you have had from participating in this intervention of telephone support?
